# Amino acid nutrition and metabolism in domestic cats and dogs

**DOI:** 10.1186/s40104-022-00827-8

**Published:** 2023-02-21

**Authors:** Peng Li, Guoyao Wu

**Affiliations:** 1 North American Renderers Association, Alexandria, Virginia 22314 USA; 2 Department of Animal Science, Texas A&M University, College Station, TX 77843 USA

**Keywords:** Animal-sourced foodstuffs, Cats, Dogs, Health, Metabolism, Nutrition

## Abstract

Domestic cats and dogs are carnivores that have evolved differentially in the nutrition and metabolism of amino acids. This article highlights both proteinogenic and nonproteinogenic amino acids. Dogs inadequately synthesize citrulline (the precursor of arginine) from glutamine, glutamate, and proline in the small intestine. Although most breeds of dogs have potential for adequately converting cysteine into taurine in the liver, a small proportion (1.3%–2.5%) of the Newfoundland dogs fed commercially available balanced diets exhibit a deficiency of taurine possibly due to gene mutations. Certain breeds of dogs (e.g., golden retrievers) are more prone to taurine deficiency possibly due to lower hepatic activities of cysteine dioxygenase and cysteine sulfinate decarboxylase. De novo synthesis of arginine and taurine is very limited in cats. Thus, concentrations of both taurine and arginine in feline milk are the greatest among domestic mammals. Compared with dogs, cats have greater endogenous nitrogen losses and higher dietary requirements for many amino acids (e.g., arginine, taurine, cysteine, and tyrosine), and are less sensitive to amino acid imbalances and antagonisms. Throughout adulthood, cats and dogs may lose 34% and 21% of their lean body mass, respectively. Adequate intakes of high-quality protein (i.e., 32% and 40% animal protein in diets of aging dogs and cats, respectively; dry matter basis) are recommended to alleviate aging-associated reductions in the mass and function of skeletal muscles and bones. Pet-food grade animal-sourced foodstuffs are excellent sources of both proteinogenic amino acids and taurine for cats and dogs, and can help to optimize their growth, development, and health.

## Introduction

The domestic dog (*Canis familiaris*) and the domestic cat (*Felis catus*) have been human companions for at least 12,000 and 9000 years, respectively [1, 2]. These animals contribute to the mental health and well-being of children, adolescents, and adults, and have become increasingly popular in many countries and worldwide over the past decades (Table 1). For example, the numbers of domestic cats and dogs in China have increased gradually between 2013 and 2022 by 677% and 147%, respectively [3]. In the United States, 25.4% and 38.4% of households owned cats and dogs, respectively, in 2018, as companions or family members [4]. Most petfoods are commercially manufactured, although some people choose to prepare meals for their own pets by using animal- and plant-sourced ingredients. Thus, the global petfood industry has grown substantially in recent years. The compound annual growth rate of the global petfood market is expected to be 4.6% between 2020 and 2027 (monetary value, US $124.9 billion by 2027) [5].

**Table 1:** Numbers of domestic dogs and cats as pets worldwide (× 10^6^)^a^

World or country	Year	Dogs	Cats
World	2018	471	373
	2020	510	400
Brazil	2010	34	18
	2018	52	22
	2020	56	23
Canada	2012	6.5	8.0
	2018	7.6	8.1
	2020	7.7	8.1
China	2013	55	22
	2017	85	69
	2018	94	87
	2022	136	171
Europe	2010	74	85
	2018	85	104
	2020	90	110
Japan	2013	10.9	9.7
	2018	8.9	9.6
	2020	8.5	9.6
India	2014	13	1.2
	2018	19	1.7
	2020	22	1.9
USA	2011	70	74
	2018	77	58
	2020	85	65

^a^ Data for the USA are taken from the American Veterinary Medical Association at https://www.avma.org/resources-tools/reports-statistics/us-pet-ownership-statistics. Data for other regions of the world are taken from Statista at https://www.statista.com/statistics

The dog is a domesticated descendant of the grey wolf (an obligate carnivore), and was from the taxonomical order Carnivora over 15,000 years ago [6]. The cat, which was also from the order Carnivora, is the only domesticated species in the family Felidae [7]. The feline domestication occurred approximately 10,000 years ago [7]. To date, about 450 dog breeds [8] and 60 cat breeds [9] with differences in shape, size, and color are recognized globally. Puberty or sexual maturity occurs in domestic cats and dogs at 6–12 and about 6 months, respectively, whereas the life span of cats and dogs is 12–18 and 10–16 years, respectively, depending on size, breed, and nutritional status. Both cats and dogs have: (a) a relatively shorter digestive tract, longer canine teeth, and a tighter digitation of molars than omnivorous mammals such as humans and pigs, (b) a very low activity of salivary α-amylase, (c) a limited ability to synthesize de novo arginine and vitamin D or to convert α-linolenic acid to 5,8,11,14,17,20-docosahexaenoic acid, and (d) instinct preferences for meat to plant products [10, 11]. Based on their anatomical, metabolic, and natural feeding characteristics, dogs (facultative carnivores) and cats (obligate carnivores) are classified as carnivores in classic animal nutrition [12] and veterinary medicine [13] textbooks, but these animals have evolved to have some unique feeding behaviors and metabolic characteristics that are distinct from omnivorous mammals such as pigs, rats, and humans [10, 14, 15]. The National Research Council (NRC) [16] recognizes that the dog is a carnivore anatomically but has many metabolic characteristics of omnivores, including the conversion of β-carotene to vitamin A, tryptophan to niacin, cysteine to taurine, and linoleic acid to arachidonic acid. Dogs also differ from cats in many of these aspects. For example, unlike cats that require much more dietary protein (expressed as % of diet) than provided in grains and vegetables, most breeds of dogs can thrive on taurine-free vegetarian diets that are properly balanced and sufficient in non-taurine nutrients through supplementation with those either absent from plants or inadequately synthesized de novo [16]. Regardless of their sources of food, adequate knowledge of nutrient metabolism and requirements by cats and dogs is crucial to ensure their optimal growth, development, and health. The major objective of this article is to highlight the nutrition and metabolism of amino acids (AAs) in cats and dogs. Unless indicated, AAs except for glycine and taurine refer to the *L*-isomers herein.

## Digestibilities of amino acids in the diets of cats and dogs

Determination of AA digestibilities in cats and dogs usually requires a 5-d period of adaptation and a 5-d period of sample collection [16]. To date, the fecal collection method has been largely used to measure the apparent total tract digestibilities of AAs in cats, and some studies have involved sample collection at the end of the ileum in dogs [17–20]. Data on total tract digestibilities of amino acids are not useful for formulating animal diets, but comparison between apparent ileal and total tract AA digestibilities may help to assess the extent of metabolism of individual AAs in the large intestine [15]. Although there are substantial amounts of endogenous AAs in the small intestine of both cats and dogs (Table 2) due to gastrointestinal secretions such as sloughed mucosa and digestive enzymes [21–24], there are no data on the true ileal digestibilities of AAs and other nutrients in these animals due to both technical and ethical challenges. Nevertheless, standardized ileal digestibilities of individual AAs based on corrections for endogenous AA flow in the ileum have been reported for dogs [15]. According to published studies [25, 26], standardized ileal digestibilities of AAs in dogs are similar to, or lower than, those for pigs (Table 3). Compared with dogs, cats generally have lower apparent digestibilities of AAs for lower-quality proteins due to a shorter small intestine relative to body weight (BW) but have similar values for high-quality proteins (e.g., those with 90% or higher apparent digestibilities) [27].

**Table 2:** Endogenous excretion of amino acids and nitrogen at the terminal ileum of adult cats, dogs, rats, and pigs

Amino acid	Adult dogs^a^		Adult cats^b^		Adult rats^a^		Pigs^c^	
	Protein-free diet	Enzyme-hydrolyzed casein	Protein free diet	Enzyme- hydrolyzed casein	Protein-free diet	Enzyme-hydrolyzed casein	15-kg BW	75-kg BW
μg/g of dry matter intake								
Ala	515	951	666	1380	269	474	437	454
Arg	370	728	540	948	159	328	480	435
Asp + Asn	960	2428	1283	2725	660	1095	755	640
Cys	–	–	452	853	–	–	–	136
Glu + Gln	1089	5993	1427	4240	696	1513	786	735
Gly	600	1001	665	1298	513	435	1660	1053
His	268	700	397	897	136	218	231	166
Ile	362	1191	398	1205	249	575	231	265
Leu	560	1180	884	1823	378	706	399	459
Lys	441	866	570	1101	193	390	312	427
Met	119	323	164	411	75	157	–	65
Phe	465	624	632	1015	171	316	237	265
Pro	643	1814	820	1913	442	691	3557	3104
Ser	925	3386	1013	2734	407	887	549	345
Taurine	–	–	2091	299	–	–	–	–
Thr	1168	2015	1235	2127	450	659	571	423
Tyr	444	648	599	1046	180	361	181	213
Val	536	1448	696	1687	273	599	321	387
mg/g of dry matter intake								
AA N	1.27	3.33	1.9	3.6	0.71	1.23	1.63	1.40
Total N	2.27	4.12	2.4	3.6	1.91	1.63	2.16	–

“–” data are not available

^a^Hendriks et al. [21]. Adult dogs and rats were fed either a protein-free diet or a diet containing 23% enzyme-hydrolyzed casein

^b^Hendriks et al. [22]. Adult cats were fed either a protein-free diet or a diet containing 14% enzyme-hydrolyzed casein

^c^The values were determined in 15-kg growing pigs [23] and 75-kg growing pigs [24] fed a protein-free diet

**Table 3:** Apparent ileal, total tract, and standardized digestibilities of amino acids in adult dogs, cats, and pigs, %

Nutrients	Adult dogs^a^			Apparent digestibility (total tract) by adult cats^b^		Pigs^c^	
	Apparent digestibility		Standardized ileal digestibility	Hair removed	Hair included	Apparent ileal digestibility	Standardized ileal digestibility
	Ileal	Total tract (fecal)					
Dry matter	75.1	81.2	–	80.1	72.0	–	–
Organic matter	79.4	85.3	–	–	–	–	–
Crude protein	76.2	81.9	81.4	82.4	73.4	80.7	87.8
Lipids	96.5	92.4	–	95.3	93.4	–	–
Carbohydrates	88.9	95.5	–	–	–	–	–
Ala	77.7	81.6	80.8	86.8	78.6	78.9	85.9
Arg	87.3	91.7	89.6	91.4	85.3	91.0	94.9
Asp + Asn	67.0	82.0	71.1	82.1	70.7	82.8	88.0
Cys	56.5	75.3	–	75.4	42.2	79.9	87.6
Glu + Gln	81.6	87.1	84.3	85.5	76.9	87.1	90.8
Gly	73.1	83.7	76.7	90.0	82.9	74.3	89.1
His	61.4	78.7	66.1	–	–	87.5	91.3
Ile	77.7	80.6	81.6	–	–	81.9	87.5
Leu	79.4	84.9	82.3	85.1	76.1	85.0	89.1
Lys	76.8	81.6	80.0	–	–	83.8	88.9
Met	82.6	83.8	85.1	–	–	86.4	90.1
Phe	80.3	84.6	84.7	–	–	85.2	89.4
Pro	78.8	88.2	82.6	–	–	80.5	104.2
Ser	69.0	82.6	78.1	84.9	72.9	84.7	90.6
Thr	62.0	79.0	75.1	83.5	72.5	78.5	86.2
Tyr	77.2	82.8	82.5	–	–	85.1	90.5
Val	72.3	79.6	76.9	–	–	79.9	86.0
AA nitrogen	76.7	84.6	80.2	85.7	76.5	83.1	90.0

*AA* Amino acid, “–” data are not available

^a^ Hendriks et al. [15]. Adult female dogs with a mean body weight of 25.3 kg, a mean age of 3 years, and a mean food intake of 389.4 g/d (15.4 g food/kg body weight/d) were fed commercial dry canine foods containing 24.3%–32.7% crude protein (dry matter basis). Apparent ileal digestibility = (AA intake – AA in ileal digesta)/AA intake × 100; Apparent total tract (fecal) digestibility = (AA intake – AA in feces)/AA intake × 100; Standardized ileal digestibility = [AA intake – (AA in ileal digesta – basal endogenous AA flow in ileum)]/AA intake × 100

^b^ Kim et al. [25]. Adult domestic cats (7 males and 7 females) with a mean body weight of 4.5 kg and a mean age of 3.3 years were fed a dry extruded diet (chicken-based, grain-free commercial feed with 2% refined cellulose and 3% sugar beet pulp) containing 33% CP. Hair of cats was removed from or included in feces for determining the apparent digestibilities of nutrients

^c^ Pigs (92-kg body weight) were fed a corn- and soybean meal-based diet [26]

The cecectomized rooster model has been used to assess the digestibilities of AAs in foodstuffs for cats and dogs [28]. Based on this assay, a very low true digestibility of glycine (e.g., only 22%) has been reported for lamb and brown rice, compared with the values of > 80% for most AAs [28], possibly due to an inaccurate measurement of endogenous glycine flow in the ileum. In addition, results from a study with a swine model indicated that the apparent ileal digestibility of cysteine and proline (i.e., 30%) in meat and bone meal as compared with the values of > 69% for most AAs [29] was likely due to analytical problems, as it is a technical challenge to determine these two AAs by using ion-exchange chromatography. Caution over data accuracy and test animal models should be taken when interpreting and using literature data on AA digestibilities for feeding companion animals.

Many of the factors that influence apparent AA digestibilities in dogs have similar effects in cats. In manufacturing pet foods, the heating of plant-sourced foodstuffs destroys trypsin inhibitors, but prolonged heating decreases the digestibilities of crude protein (CP) and AAs (e.g., Lys and Arg) due to the Maillard reaction [16]. Cooking can result in improved digestibilities of AAs in dogs [30] and of starch in cats [31]. Increasing the dietary level of soluble fiber reduces CP digestibility in both dogs [32, 33] and cats [31]. In contrast, dietary supplementation with *yucca schidigera* (60–120 mg/kg) enhances the digestibility and absorption of AAs in these animals, thereby reducing the odor of pet stools [34]. Extrusion of meat and bone meal via high temperature and pressure (135 °C, 3 bar, 20 min) increases the digestibility of dietary CP in adult cats but had no effect in adult dogs [27], indicating species differences in nutrient digestion.

The apparent ileal and total tract digestibilities of CP in diets containing both plant ingredients (primarily wheat and corn grains) and meat (lamb meal, poultry meal, or fish meal) were 74% and 84%, respectively, in adult dogs [35]. The apparent ileal digestibilities of individual AAs in this canine diet (%) were: Ala, 80.5; Arg, 85.1; Asp + Asn, 52.0; Cys, 54.5; Glu + Gln, 82.2; Gly, 76.2; His, 76.0; 4-hydroxyproline, 79.5; Ile, 78.7; Leu, 80.2; Lys, 78.9; Met, 82.0; Phe, 81.9; Pro, 81.5; Ser, 70.5; Thr, 69.7; Tyr, 77.4; and Val, 77.8 in the mixed breed Alaskan Husky [35]. 4-Hydroxyproline is converted into glycine via the 4-hydroxyproline oxidase pathway in animal tissues [36]. For comparison, the apparent total tract digestibilities of individual AAs in this canine diet (%) were: Ala, 87.3; Arg, 90.2; Asp + Asn, 78.3; Cys, 71.0; Glu + Gln, 88.9; Gly, 88.3; His, 86.9; 4-hydroxyproline, 94.4; Ile, 85.1; Leu, 86.8; Lys, 83.8; Met, 86.1; Phe, 86.4; Pro, 89.9; Ser, 82.3; Thr, 82.6; Tyr, 84.5; and Val, 84.5 in these dogs [35]. Similar results were reported for adult German shepherd dogs fed plant (extruded wheat, corn meal, soybean meal, and beet pulp)- and animal (fishmeal, poultry meal, meat meal, and meat & bone meal)-based diets containing 30% CP, 19% lipids, 29%–34% starch, and 6%–13% fiber [37], and for adult dogs fed commercial dry foods (Table 3). Comparisons between the apparent ileal and total tract digestibilities of methionine and lysine in dogs indicate that, in contrast to pigs [38], (a) a substantial amount of protein metabolites is absorbed by the canine large intestine and (b) the microbes of the canine large intestine do not have a net synthesis of methionine and lysine. These findings suggest an important difference in gut microbial metabolism among animal species.

Digestibilities of AAs are influenced by the breed and age of dogs, as well as diet and the method of its preparation. For example, at 11, 21, 35, and 60 weeks of age, apparent CP digestibilities were greater in large breeds than small breeds of dogs [39] and were increased with age, possibly due to a greater activity of intestinal microbes. The apparent digestibility of CP was reduced by 5% in 11-week-old puppies compared with 2- to 4-year-old adult dogs, but no differences were detected between 0.5- and 2-year-old dogs [40]. Likewise, no difference in CP digestibility was noted between 2- and 17-year-old beagles [41]. Dietary supplementation with β-mannanase (the enzyme hydrolyzing polysaccharides made from *D*-mannose) enhanced the apparent digestibilities of CP in dogs fed a diet containing a large amount of plant-sourced protein ingredients, but had no effect in dogs fed a diet containing a large amount of animal-sourced protein ingredients [42]. Compared with the extruded diet, slight cooking enhanced CP digestibility in dogs [30]. In adult dogs, increasing the content of dietary CP from 18% to 42% did not affect the standardized ileal digestibilities of AAs [17], indicating a high ability of these animals in digesting dietary protein. Likewise, the inclusion of 7.5% fiber in diets did not adversely affect the apparent ileal digestibilities of CP and AAs in adult dogs [30], further supporting the notion that these animals can adapt well to an appropriate proportion of plant-sourced ingredients in their diets [43].

Hair, which is lost from the skin, should be removed from feces for accurately measuring nutrient digestibility in cats [25]. Harper and Turner [44] reported that 19-week-old cats had higher apparent CP digestibilities than younger kittens. In adult cats, the apparent digestibilities of CP were 91%–94%, 87%–88%, or 89%–90%, respectively, in a meat (a mixture of beef and mutton)-based diet containing 22% cornstarch, 14.3% corn, or 14.3% wheat grains, and were not affected by fine grinding or cooking [31]. Likewise, the apparent digestibilities of CP by healthy adult cats did not differ between two diets containing 36%–55% high-quality proteins (a high proportion of meat) and 37%–56% low-quality proteins (a low proportion of meat) (i.e., 89%–90% versus 88%–91%, respectively) [45], but most likely did not reflect the true digestibilities of the proteins due to microbial AA metabolism in the large intestine. However, the apparent digestibility of dietary CP was decreased in cats fed raw corn starch and raw potato starch compared with cooked foods [46]. These results indicate effects of age and dietary composition on the digestion of dietary protein and microbial AA metabolism in the gut.

## Metabolism of AAs by cats and dogs

The metabolism of most AAs by cats and dogs is similar to that of other mammals [43]. In support of this view, the plasma concentrations of most AAs from different research groups [47–50] are similar between adult cats and dogs, except for Asn, Asp, Citrulline, Glu, Gly, His and Pro (that are lower in dogs than in cats) and for lysine (that is higher in dogs than in cats) (Table 4). Interestingly, the plasma concentrations of Arg, citrulline, and ornithine in both cats and dogs were lower than those for pigs [51, 52] (Table 4), suggesting differences in the whole-body metabolism of AAs among the three animal species.

**Table 4:** Concentrations of amino acids (AAs) in the plasma of dogs, cats, and pigs

AAs	Dogs^a^				Cats		Pigs		
	< 2 wk of age (*n* = 7)	2 wk to 2 mo of age (*n* = 7)	12 to 60 mo of age (*n* = 7)	> 60 mo of age (*n* = 8)	Young kittens^c^ (*n* = 8)	Adults^d^ (*n* = 120)	4 d of age^e^ (*n* = 10)	2.5 mo of age^f^ (*n* = 8)	18 mo of age^g^ (*n* = 8)
Ala	315 ± 46	296 ± 180	400 ± 129	332 ± 162	603 ± 62	462 ± 160	1049 ± 478	596 ± 91	352 ± 58
Arg	278 ± 80	245 ± 101	103 ± 29	104 ± 36	–	95 ± 38	163 ± 63	159 ± 45	165 ± 19
Asn	121 ± 43	121 ± 48	52 ± 16	47 ± 10	–	91 ± 25	118 ± 28	62 ± 8	66 ± 10
Asp	19 ± 9	11 ± 3	trace	trace	–	28 ± 12	25 ± 9	13 ± 6	12 ± 5
Cit	88 ± 39	107 ± 45	34 ± 10	38 ± 12	–	18 ± 6	89 ± 25	64 ± 6	60 ± 8
Cys^h^	42 ± 38	56 ± 36	32 ± 22	42 ± 26	–	26 ± 9	165 ± 33^g^	173 ± 12	170 ± 23
Gln	564 ± 174	461 ± 139	593 ± 175	658 ± 213	–	664 ± 134	469 ± 120	513 ± 79	491 ± 59
Glu	112 ± 82	73 ± 32	35 ± 8	38 ± 12	–	73 ± 38	156 ± 63	172 ± 40	92 ± 15
Gly	283 ± 85	364 ± 107	181 ± 61	171 ± 42	–	398 ± 279	824 ± 237	664 ± 62	688 ± 81
His	140 ± 45	85 ± 28	66 ± 13	65 ± 15	–	116 ± 24	114 ± 47	103 ± 20	97 ± 16
Ile	83 ± 24	70 ± 23	67 ± 21	63 ± 18	51 ± 8	63 ± 29	159 ± 57	112 ± 28	104 ± 15
Leu	212 ± 39	147 ± 69	142 ± 34	135 ± 48	91 ± 14	146 ± 49	186 ± 51	246 ± 42	196 ± 18
Lys	275 ± 68	195 ± 121	149 ± 46	191 ± 55	–	108 ± 61	223 ± 82	88 ± 48	103 ± 35
Met	52 ± 33	73 ± 20	46 ± 15	52 ± 16	116 ± 51	64 ± 28	100 ± 51	35 ± 6	47 ± 6
Orn	85 ± 31	43 ± 16	19 ± 9	17 ± 7	–	21 ± 12	107 ± 38	85 ± 17	82 ± 20
Phe	60 ± 13	64 ± 25	61 ± 14	63 ± 18	72 ± 11	70 ± 15	117 ± 35	87 ± 11	89 ± 14
Pro	389 ± 106	290 ± 105	145 ± 61	114 ± 15	–	258 ± 76	628 ± 373	395 ± 59	382 ± 50
Ser	241 ± 40	262 ± 91	123 ± 28	138 ± 31	–	179 ± 85	267 ± 123	153 ± 45	157 ± 38
Taurine	–	–	–	77 ± 24^b^	–	118 ± 55	169 ± 57	56 ± 17	60 ± 21
Thr	523 ± 245	250 ± 151	196 ± 63	157 ± 50	168 ± 51	173 ± 54	369 ± 145	89 ± 45	93 ± 32
Trp	66 ± 27	55 ± 15	69 ± 27	54 ± 17	–	60 ± 17	38 ± 19	47 ± 8	45 ± 9
Tyr	88 ± 31	54 ± 24	48 ± 7	43 ± 8	36 ± 8	57 ± 15	242 ± 73	105 ± 17	112 ± 24
Val	248 ± 42	200 ± 83	199 ± 43	199 ± 63	131 ± 34	164 ± 62	350 ± 114	280 ± 37	216 ± 31

*Cit* Citrulline, *mo* months, *Orn* Ornithine, *Tau* Taurine, *wk* weeks, “–” data were not available

Values are means ± SD

^a^ Blazer-Yost and Jezyk [47] for all amino acids except taurine

^b^ Delaney et al. [48]. Adult cats (*n* = 131) had a median age of 5.3 years (ranging from 2 to 14 years)

^c^ Hargrove et al. [49]. Kittens (1 to 2 kg body weight) were fed a casein-, soy protein-, and AA mix-based diet

^d^ Heinze et al. [50]

^e^ Flynn and Wu [51]

^f^ Wu et al. [52]

^g^ Wu G (unpublished work). Adult gilts were fed, twice daily at 1% of body weight per meal, a corn- and soybean meal-based diet containing 12.2% CP [53]. Blood samples were obtained from the jugular vein at 2 h after feeding to prepare plasma for AA analysis [52]

^h^ cysteine + ½ cystine

The qualitative dietary requirements of dogs for most AAs are similar to those for omnivores (e.g., humans and pigs) [16, 53]. However, in contrast to most breeds of dogs, cats have a very limited ability to synthesize taurine and arginine [14]. Because taurine is present in animal products but absent from plants [54], cats must be provided with at least a portion of animal-sourced foods or the same essential nutrients from synthetic supplements [43]. This is consistent with the much greater concentrations of both taurine and arginine in the milk of cats [55–58] as compared with ruminants and pigs (Table 5) [59–61]. Thus, there are peculiar differences in the requirements of certain AAs, such as taurine (essential for tissue integrity) [62] and arginine (essential for maintaining the urea cycle in an active state) [63] between cats and dogs. Likewise, the concentrations of many AAs in plasma differ between cats and dogs offered diets high in carbohydrate, high in fat, or high in protein [64]. Cats and dogs chose a different mix of food, which is consistent with cats needing a higher protein concentration in food than dogs [64]. In these two animal species, the synthesis of glucose from AAs in the liver and kidneys plays an important role in maintaining glucose homeostasis [43]. When diets do not provide sufficient starch, glycogen or glucose, dogs must synthesize glucose from glucogenic AAs in their liver and kidneys [65]. In contrast to modern breeds of dogs that consume both animal- and plant-sourced foods, a natural food (i.e., meat) for cats contains only a small amount of glycogen (primarily from muscle and liver; < 5%, DM basis) and no starch [14]. Thus, in cats, gluconeogenesis from AAs plays an essential role in the provision of glucose to the brain, red blood cells, and immunocytes, and therefore their survival [12].

**Table 5:** Concentrations of total amino acids (free plus peptide-bound) in the mature milk of cats, dogs, cows, goats, and pigs

Nutrient	Dogs^a^ (*n* = 16)	Cats^b^ (*n* = 4)	Cows^b^ (*n* = 4)	Goats^c^ (*n* = 30)	Pigs^f^ (*n* = 10)
Water, g/kg milk	773	790	877	870	799
Dry matter, g/kg milk	227	210	123	130	201
Crude protein, g/kg milk	75	75	33	35	48
Total amino acids, g/L milk					
Ala	2.50	2.80 ± 0.5	1.08 ± 0.03	1.18	1.97 ± 0.06
Arg	2.93	4.85 ± 0.5	1.14 ± 0.03	1.36	1.43 ± 0.08
Asp + Asn	9.53	6.51 ± 2.0	2.35 ± 0.17	2.51	5.12 ± 0.12
Cys	2.48	0.91 ± 0.5	0.30 ± 0.03	0.31	0.72 ± 0.05
Glu + Gln	8.90	15.8 ± 0.5	6.99 ± 0.07	6.95	9.44 ± 0.37
Gly	1.56	0.76 ± 0.5	0.61 ± 0.03	0.56	1.12 ± 0.07
His	1.35	2.04 ± 0.5	0.81 ± 0.03	1.23	0.92 ± 0.05
4-Hydroxyproline	–	–	0.48 ± 0.04^f^	–	0.82 ± 0.04
Ile	1.97	3.26 ± 0.5	1.58 ± 0.03	1.61	2.28 ± 0.10
Leu	5.48	8.93 ± 0.5	3.33 ± 0.03	3.41	4.46 ± 0.17
Lys	3.17	4.32 ± 0.5	2.89 ± 0.07	3.43	4.08 ± 0.15
Met	1.41	2.42 ± 0.5	0.87 ± 0.03	0.78	1.04 ± 0.04
Phe	3.53	2.27 ± 0.5	1.68 ± 0.03	1.76	2.03 ± 0.11
Pro	3.62	7.12 ± 1.0	3.36 ± 0.13	3.11	5.59 ± 0.26
Ser	3.26	3.33 ± 0.5	1.88 ± 0.03	1.53	2.35 ± 0.11
Thr	3.44	3.48 ± 0.5	1.41 ± 0.03	1.39	2.29 ± 0.14
Trp	0.26	–	0.43 ± 0.02^e^	–	0.66 ± 0.02
Tyr	3.57	3.41 ± 0.5	1.58 ± 0.03	1.63	1.94 ± 0.05
Val	3.80	3.56 ± 0.5	1.75 ± 0.03	2.10	2.54 ± 0.09
Taurine	0.33 ± 0.14^d^	0.36 ± 0.04^d^	0.007 ± 0.001^f^	0.098^e^	0.19 ± 0.013^f^

Adapted from Rezaei et al. [55] for the content of water, dry matter, and crude protein in milk. Values for total amino acids were calculated on the basis of their intact molecular weights, and are expressed as either means or means ± SEM when data are available

^a^ Ferrando et al. [56] for proteinogenic amino acids

^b^ Davis et al. [57] for proteinogenic amino acids

^c^ Ceballos et al. [58] for proteinogenic amino acids

^d^ Rassin et al. [59]

^e^ Prosser [60]

^f^ Wu [61]

### Endogenous nitrogen excretion

In adult dogs (BW ranging from 2.8 to 51 kg) fed a protein-free, semi-purified diet, the outputs of endogenous urinary nitrogen, metabolic fecal nitrogen [nitrogen originating from the sloughed gastrointestinal epithelium and bacteria, as well as other endogenous sources (e.g., saliva, mucus, bile, and pancreatic and intestinal secretions)], and total endogenous nitrogen were 210, 63, and 273 mg/kg BW^0.75^/d, respectively [66]. This is equivalent to the catabolism of 1.71 g protein/kg BW^0.75^/d. There was no significant effect of either sex or BW on the measured variables expressed per metabolic BW, but endogenous urinary nitrogen output was positively correlated with BW loss during the 14-d feeding period [66]. The maintenance requirement of adult dogs for CP is 82 g/kg of the diet containing 4.0 kcal ME/g diet, and the NRC-recommended allowance for CP is 100 g/kg of the diet containing 4.0 kcal ME/g (DM basis) [16].

The endogenous excretions of total, urea, ammonia, and creatinine (a metabolite of creatine) nitrogen for cats fed the protein-free diet were 360, 243, 27.6, and 14.4 mg/kg BW^0.75^/d, respectively [67]. Similarly, Earle [68] reported that adult cats could maintain nitrogen balance or had minimal endogenous nitrogen loss at 1.4–1.7 g protein/kg BW/d. These values are greater than those for dogs and pigs (Table 2). For comparison, the rates of urinary nitrogen excretion (mg nitrogen/kg BW^0.75^/d) when fed a nitrogen-free diet were: human, 62; marmoset, 110; rat, 128; pig, 163; dog, 210; and cat, 360 [16]. Accordingly, adult cats require 2 to 3 times more dietary protein than adult dogs and herbivores (e.g., cows, sheep, and horses) [14]. The maintenance requirement and the recommended allowance of dietary CP by the adult cat are 160 and 200 g/kg diet containing 4.0 kcal ME/g diet (DM basis), respectively [16]. This is equivalent to the minimum maintenance requirement of adult cats for dietary protein energy (16% of dietary ME). For comparison, a dietary intake of protein energy accounting for 3.5%–4.5% of dietary ME is sufficient to maintain BW, nitrogen balance, and carcass nitrogen content in adult rats [69, 70]. The obligatory loss of nitrogen in cats appears to be similar when they are fed a nitrogen-free diet or are food deprived [16]. Interestingly, nitrogen balance in adult cats fed a low-protein diet may be maintained when lean body mass is reduced [71], possible due to reduced oxidation of AAs in a tissue-specific manner [61]. This must be taken into consideration when determining AA requirements of cats.

### Metabolism of arginine

There have been many studies of arginine nutrition in dogs since the pioneering work of Rose and Rice in 1939 [72]. Dogs can synthesize arginine from dietary glutamine/glutamate and possibly dietary proline, as well as arterial glutamine via the intestinal-renal axis [63]. In adult dogs [73, 74], as in many other adult mammals (e.g., humans, pigs, rats, and sheep) [61], the small intestine synthesizes and releases citrulline, which is taken up by extraintestinal tissues (primarily the kidneys) for arginine synthesis. The small intestine and other organs of dogs express arginase for the hydrolysis of arginine to urea and ornithine [75].

In adult dogs, the activity of arginase (expressed on the basis of tissue protein) is similar between the duodenum and jejunum, with values for the ileum being 24%–37% of those for the upper parts of the small intestine [75]. Unlike pigs [61], the small intestine of postabsorptive dogs does not release arginine [73], likely due to either low activities of argininosuccinate synthase and lyase  for arginine synthesis or the further hydrolysis of arginine by arginase in enterocytes. Thus, the homeostasis of arginine in the body depends on the rates of its endogenous synthesis and catabolism. Growing and adult dogs cannot synthesize sufficient arginine to meet functional needs (e.g., ammonia detoxification via the urea cycle) beyond maintaining nitrogen balance [43]. Thus, a dietary level of 0.4% and 0.28% arginine is needed for the maximum growth of young dogs and the hepatic ureagenesis in adult dogs, respectively, if other AAs are sufficient [43]. This indicates that both mature and immature dogs have an inadequate or limited ability to synthesize arginine de novo. Syndromes of arginine deficiency in dogs include decreased food intake, hyperammonemia, severe emesis, frothing at the mouth, and muscle tremors, and can be prevented by dietary supplementation with arginine or citrulline [16]. Dietary or arterial blood ornithine is not used for arginine synthesis and cannot correct arginine deficiency symptoms in dogs [43]. Interestingly, dog’s milk contains much more arginine than the milk of herbivores (e.g., cows) and omnivores (e.g., humans and pigs) [76] to ensure that canine neonates receive adequate arginine for survival and growth. When fed a milk-replacer diet containing inadequate arginine, dog puppies develop cataract [77]. Dietary arginine deficiency also occurs in human infants (causing hyperammonemia and death) and adults [reducing nitric oxide (NO) synthesis, sperm production, and fetal growth], and in rats (impairing growth and spermatogenesis) [61].

Cats have a very limited ability to synthesize citrulline and arginine de novo because of the low activities of pyrroline-5-carboxylate (P5C) synthase and ornithine aminotransferase [78]. The latter also limits the formation of citrulline from proline via the proline oxidase pathway. There is evidence for the synthesis of arginine from citrulline and the catabolism of arginine via arginase in feline renal tubules [79]. In cats, when dietary intake of arginine is insufficient, food ingestion is reduced, followed by hyperammonemia (occurring within 1–3 h after feeding) due to impaired ureagenesis in the liver, vomiting, neurological signs, severe emesis, ataxia, tetanic spasms, and death [80]. Dietary supplementation with citrulline or ornithine to cats can prevent hyperammonemia due to arginine deficiency. However, citrulline, but not ornithine, can restore growth in young cats fed an arginine-free diet [80]. Such results can be explained by the findings that dietary or arterial blood ornithine is not used for the intestinal synthesis of citrulline in cats [63], as reported for other mammals including dogs [73] and pigs [53]. This is due to both the preferential metabolism of dietary ornithine into proline by enterocytes and the lack of uptake of arterial blood ornithine by the gut [63]. We suggest that higher protein requirements by cats than dogs may result, in part, from a much lower ability to synthesize arginine in cats. The sensitivity of mammals to dietary arginine deficiency is cats > dogs > rats [14].

### Metabolism of aspartate, glutamate, and glutamine

In canine and feline nutrition, aspartate, glutamate, and glutamine are among the traditionally classified nutritionally nonessential AAs (NEAAs), but this has been disputed [81]. Mammals, including dogs, use dietary aspartate, glutamate, and glutamine as well as arterial glutamine as the major metabolic fuels in their small intestine [82], but cannot adequately synthesize aspartate, glutamate, and glutamine [75]. Both aspartate and glutamate are essential for intestinal metabolism, but are not taken up by the small intestine from the arterial blood [61]. Thus, these two AAs are required in cat and dog diets.

There is a large database on the metabolism of the glutamine family of AAs in the small intestine [75] and kidneys [83] of dogs. Dietary aspartate, glutamate, and glutamine are extensively degraded in the mucosa of the canine small intestine as metabolic fuels [82]. Glutamine, but not aspartate and glutamate, in the arterial blood, is taken up by the canine small intestine for metabolism [73]. The small intestine accounts for ~ 30% of the arterial blood glutamine utilized by healthy adult dogs, and release ammonia, alanine, proline, and citrulline but little or no ornithine and arginine [84–86]. This involves the conversion of glutamine into alanine, proline and citrulline via a series of enzymes including glutaminase, glutamate transaminases, P5C synthase, and P5C reductase [61]. In addition, glutamine is the major source of glutamate for gluconeogenesis and ammoniagenesis for the regulation of acid-base balance in the canine kidneys [87]. The utilization of arterial glutamine by the small intestine of dogs is increased by ~ 80% during treadmill exercise due to elevated concentrations of glucagon, leading to a 17% decrease in plasma glutamine concentration [88]. Likewise, an intraluminal infusion of glucose, which stimulate the release of glucagon from the pancreas, can enhance the uptake of arterial blood glutamine and the release of ammonia, alanine, glutamate, and citrulline by the small intestine of dogs [89]. In response to metabolic acidosis, the uptake of glutamine by the canine kidneys is markedly increased to meet the demand for renal ammoniagenesis. Interestingly, in contrast to rats [82], the extraction of glutamine by the small intestine of dogs is increased during progressive fasting (up to 4 d) via unknown biochemical mechanisms [90], indicating another species difference between dogs and omnivorous mammals in the regulation of intestinal glutamine metabolism in response to food deprivation.

Like dogs, the small intestine of cats takes up arterial blood glutamine and releases ammonia [82]. In the fasted state, the feline small intestine extracts ~ 20% of glutamine from the arterial blood [86]. Little is known about the metabolism of other AAs in the small intestine of cats. Besides published data on arginine synthesis from citrulline and the catabolism of arginine via arginase in renal tubules [79], there are no reports on the metabolism of other AAs in the feline kidneys. We are not aware of studies to compare the tissue-specific- or whole-body metabolism of aspartate, glutamate, and glutamine among animal species (including cats).

### Metabolism of branched-chain AAs (BCAAs)

Dietary BCAAs (~ 30%) are extracted by the small intestine of fed dogs in first-pass metabolism, with 55% and 45% of the utilized leucine entering the transamination and protein synthesis pathways, respectively [91]. As reported for rats, the liver of dogs does not degrade BCAAs [92] due to the near absence of BCAA transaminase in hepatocytes. Rates of BCAA uptake by extrahepatic tissues determine the availability of these nutrients for metabolic utilization. In fasted dogs (20 kg BW; a food deprivation period of ~ 36 h), skeletal muscle takes up leucine from the arterial blood at the rate of 0.89 μmol/kg BW/min [93]. Based on the uptake of total BCAAs (0.143 μmol/kg BW/min) by the skeletal muscle of 12 h-fasted pigs (20–25 kg BW) [94] and the ratio of leucine:isoleucine:valine (0.32:0.23:0.45) in the plasma of 12-h fasted pigs [75], it can be estimated that the skeletal muscle of pigs fasted for ~ 12 h takes up leucine from the arterial blood at the rate of 0.046 μmol/kg BW/min. It remains to be determined whether the reported large discrepancy in leucine uptake by skeletal muscle between dogs and pigs results from differences in animal species, age, nutritional state, and research methodology. In dogs and other mammals, most of the diet-derived BCAAs bypass the liver and are used by extra-hepatic tissues (mainly skeletal muscle) for the synthesis of alanine and glutamine in the presence of α-ketoglutarate and ammonia [92, 93]. The ammonia is derived from the blood as well as the intramuscular catabolism of purines and AAs [61]. In the post-absorptive state, alanine and glutamine account for about 50% of the AAs released from the skeletal muscle of dogs, pigs, rats, and humans [61].

At present, little is known about BCAA metabolism in the small intestine and other tissues of cats. However, increasing the dietary protein content from 15% to 65% of dietary ME increases the whole-body oxidation of leucine and urea production in adult cats by about 3 times, as measured with [1-^13^C]leucine and [^15^N_2_]urea [95]. This finding indicates that cats can adapt well to dietary AA intake through modulating AA oxidation. In contrast to dogs, cats, like other Felidae species, use leucine and valine to synthesize isovalthine and isobuteine, respectively, with hitherto unknown physiological function [96, 97]. We are not aware of studies to compare tissue-specific or whole-body BCAA metabolism among animal species (including cats).

### Metabolism of methionine, cysteine, and taurine

In the liver of dogs, methionine is catabolized to cysteine and then to taurine, but neither taurine nor cysteine is converted to methionine [14]. The rate of oxidation of cysteine to taurine depends on the dietary intakes of sulfur-AAs. Methionine is often the first or the second (after lysine) most limiting AA in plant-based diets for dogs. Dietary cysteine can replace up to 50% dietary methionine in these animals [43].

Most breeds of dogs can synthesize sufficient taurine when fed a methionine- and cysteine-adequate diet [43]. However, an inadequate intake of methionine and cysteine in diets [e.g., plant (e.g., peas, lentils, and rice)-based foods with no or insufficient taurine] may contribute to the development of dilated cardiomyopathy characterized by thin heart muscle and enlarged chambers in some breeds of dogs [98, 99]. For example, a small proportion (1.3%–2.5%) of Newfoundland dogs fed commercially available diets that were considered to be complete and balanced in nutrition have a deficiency of taurine [100] due to reduced taurine synthesis possibly as a result of gene mutations [101]. When fed protein-restricted diets, certain breeds of dogs (e.g., golden retrievers) are more prone to taurine deficiency and the development of dilated cardiomyopathy even when fed meat-based diets due to a combination of factors, including complex interactions among dietary, metabolic, and genetic factors [102]. This disorder may result from low activities of cysteine dioxygenase and cysteine sulfinate decarboxylase, as well as a limited availability of cysteine. An ability of some breeds of dogs to form taurine does not necessarily mean that they do not require dietary taurine for optimum health. Only in the breeds of dogs that possess sufficient enzymes for taurine synthesis can the adequate provision of methionine plus cysteine in their diets prevent metabolic diseases such as dilated cardiomyopathy. Although concentrations of taurine in plasma and skeletal muscle reflect its availability in these animals, those in the whole blood may not be a sensitive indictor of taurine depletion caused by a low intake of bioavailable sulfur AAs in dogs, especially in large dogs [99].

Methionine is generally the most limiting AA for cats fed a meat-based conventional diet [16]. The feline liver can convert methionine into cysteine. However, young and adult cats have a limited ability to synthesize taurine from cysteine due to low activities of cysteine dioxygenase and cysteinesulfinic acid decarboxylase; therefore, they have a requirement for dietary taurine [43]. In these animals, taurine deficiency results in dilated cardiomyopathy, heart failure, central retinal degeneration, blindness, deafness, and poor reproduction [103]. Thus, all foods for cats must include sufficient taurine.

Cats synthesize felinine, isovalthine, and isobuteine from cysteine plus acetyl-CoA, cysteine plus isovaleryl-CoA (a metabolite of leucine), and cysteine plus isobutyryl-CoA (a metabolite of valine) as unique sulfur-containing AAs [96, 97]. In intact adult male cats, the rates of urinary excretion of felinine and isovalthine are 122 and 1.8 μmol/kg BW/d, respectively [96]. At present, there are no quantitative data on the urinary excretion of isobuteine by cats. The direct source of cysteine for these synthetic pathways is glutathione. Felinine, isovalthine, and isobuteine may serve as pheromones in cats for the purpose of territorial marking, intra-species communications, and chemical signals to attract females [104]. Indeed, males produce 239% more felinine than females (122 vs. 36 μmol/kg BW/d) [105]. In addition, it is possible that the production of unique cysteine metabolites (non-toxic, non-reactive, and relatively stable) helps to prevent excessive formation of toxic and acidic substances (e.g., H_2_S, SO_2_, and H_2_SO_4_) in cats [61].

Cysteine is the most abundant AA (accounting for ~ 16% of total protein) in the hair of cats and dogs [43]. During the growth period, these animals have greater dietary requirements for cysteine than other mammals with fewer hair in the skin (e.g., pigs and humans) [61]. Although sulfur AA restriction has been reported to improve health (e.g., delayed aging and longer lifespans) in adult rodents by altering the intestinal microbiome profile [106], we are not aware of such studies with cats or dogs.

### Metabolism of other AAs

The mucosa of the canine intestine does not degrade threonine (the major AA in mucins), phenylalanine, tyrosine, or tryptophan, and its ability to catabolize or interconvert glycine and serine is very limited [85, 107], but these AAs are degraded in the canine liver [108]. Thus, when the canine small intestine is intraluminally infused with each of these AAs (10 mmol/L), there is no production of ammonia by the gut [107]. Less than 3% of glycine absorbed by the canine jejunum intraluminally infused with 10 mmol/L glycine is released as serine [107], suggesting either a low activity of serine hydroxymethyltransferase or an insufficient availability of N^5^,N^10^-methylenetetrahydrofolate as a methyl group donor in the intestinal tissues. In postabsorptive dogs, there is no release of glycine or serine by the small intestine [73], indicating the lack of their synthesis under this nutritional condition.

Aromatic AAs are utilized via multiple metabolic pathways in animals, including dogs [108–110]. Interestingly, dogs require at least twice as much phenylalanine plus tyrosine for maximal black hair color (adequate eumelanin in hair) as for growth [108, 110]. Furthermore, supplementation with tryptophan (0.145% of diet, the precursor of serotonin) can reduce territorial aggression in dogs fed a low (19%)-CP diet (with the basal tryptophan content of 0.18%) but has no effect in dogs fed a high (31%)-CP diet (with the basal tryptophan content of 0.24%) [111]. In addition, Pereira [112] found that dietary supplementation with tryptophan (12.5 mg/kg BW/d) reduced bark and stare behavior in multi-housed dogs. At present, we are not aware of studies regarding proline catabolism in the intestine and other tissues of dogs.

Little is known about the catabolism of glycine, proline, serine, threonine, phenylalanine, tyrosine, or tryptophan in the feline intestine. However, these AAs are degraded in the liver of cats [16]. As for dogs, the maintenance of adequate eumelanin in the hair of cats requires twice as much phenylalanine plus tyrosine as for whole-body growth [109]. As a precursor of serotonin (a neurotransmitter and antioxidant), tryptophan along with α-casozepine (a bioactive peptide originating from the S1 casein protein in cow’s milk) relieved anxiety while alleviating stress and aggression in cats [113]. Dietary supplementation with tryptophan (12.5 mg/kg BW/d) reduced vocalization, agonistic behavior, exploring, scratching, and agonistic interactions in multi-housed cats [114]. Interestingly, unlike omnivores, hepatic tryptophan 2,3-dioxygenase [the enzyme oxidizing tryptophan to N-formylkynurenine (the immediate precursor of kynurenine)] is not induced by glucocorticoids in cats [14]. This indicates another species difference in the regulation of AA metabolism between cats and other mammals.

### AA imbalances and antagonisms

AA imbalances (improper ratios of AAs) occur in dogs fed commercial plant-based diets containing either a small amount of or no animal-sourced ingredients (e.g., meat) [98, 102]. This is largely because most plant proteins (particularly those in cereals) are deficient in some EAAs (particularly lysine, tryptophan, threonine, methionine, and cysteine), as well as glycine and proline (the first and second most abundant AAs in the animal body) [54]. For AAs with similar chemical structures (e.g., BCAAs) or net electric charges (e.g., arginine and lysine), their improper ratios often result in antagonisms because they share the same transporters in the cell membrane for absorption and uptake, the same enzymes for catabolism, and/or the same inhibitors of their respective pathways [61]. Effects of dietary AA imbalances in dogs are more similar to those in rats than in cats [14]. For example, in response to diets deficient in one or two EAAs, the food intake of dogs is not decreased as rapidly and severely as that for cats. Inclusion of adequate animal-sourced foodstuffs or dietary supplementation with deficient AAs may aid in preventing and correcting AA imbalances and antagonisms in canine nutrition.

Based on changes in hepatic activities of AA-catabolic and urea-cycle enzymes as well as blood and urine nitrogenous metabolites, cats are less sensitive to imbalances of most AAs (except for arginine, methionine, and lysine) in diets than dogs, rats, chicks, and pigs [14]. This is because, compared with many omnivores, cats have a lower ability to sense protein-free or protein-deficient (e.g., 6% protein) diets and the diets containing excess protein (e.g., 63% protein) or most AAs, even if they exhibit protein malnutrition, lose weight, and grow poorly [115, 116]. However, cats select for or against some proteins (e.g., casein and soy protein) [115] and the solution of some AAs [116], possibly based on their chemical properties including taste. For example, in contrast to omnivorous mammals (e.g., rats and pigs), cats do not select a diet containing adequate (0.6%) threonine versus a threonine-free diet [116]. In addition, only a mild AA antagonism is exhibited by cats when fed a diet with isoleucine or valine as the limiting AA in the basal diet, and these animals do not avoid a diet containing a highly excessive amount of leucine (e.g., 10% of leucine in diet) [117]. Thus, cats are less sensitive than omnivores to leucine-isoleucine and valine antagonisms. Furthermore, a dietary deficiency of arginine greatly reduces food intake by cats [80], likely because of neurological disorders induced by hyperammonemia and reduced nitric-oxide availability [61]. Interestingly, Rogers et al. [116] reported that when adult cats were offered diets containing either 0 or 0.2% methionine, they initially ate only the methionine-containing diet, but beginning on d 3, they consumed an increasing amount of the methionine-free diet, and on d 6 selected the same amount of each diet. It is unknown why cats select for methionine, but this may be because of its bitter taste, just like leucine. Finally, although cats select for 0.5 to 50 mmol/L lysine (prepared in saline) over saline [118], increasing the dietary content of lysine from 1.1% to 3.6%, 6.1%, and 8.6% in a diet containing 1.3% arginine and 4.09 kcal ME/g diet does not result in lysine-arginine antagonism in adult cats [119]. Further increasing the dietary content of lysine to 11.1% and 13.1% gradually reduced the food intake of adult cats by 30%–35% without affecting health [119]. Such a severe imbalance between lysine and arginine would be detrimental for omnivores and herbivores [61].

### Metabolic adaptation to low or high protein intakes

Most omnivores (e.g., rats and pigs) and herbivores (e.g., sheep and cattle) can adapt to: (1) low-protein diets by increasing food intake and reducing AA catabolism, and (2) high-protein diets by initially reducing food intake for 1–5 d (depending on dietary protein levels), followed by up-regulating the expression and/or activities of AA-catabolic enzymes [61]. For example, rats can down- or up-regulate AA-catabolic and urea-cycle enzymes in response to a low or high intake of AAs [14]. In contrast, low protein intake did not affect whole-body protein degradation in the postabsorptive state, and increasing dietary protein intake from 32 g CP/Mcal ME (low intake) to 63 g CP/Mcal ME (medium intake) and to 148 g CP/Mcal ME (high intake) did not affect the rate of leucine oxidation in adult dogs under these nutritional conditions [120]. Thus, dogs may not be as efficient as rats in metabolic adaptation to low or high protein intakes. Experimental evidence shows that dogs can tolerate at least 30%–32% dietary protein [111, 121].

Adult cats have a 100% greater requirement for dietary protein than do adult dogs, but dietary requirements for some EAAs do not appear to differ appreciably between these two animal species [16]. This may be explained by higher requirements of cats for NEAAs to fulfill metabolic needs than do dogs [61]. First, dietary NEAAs (particularly aspartate, glutamate, and glutamine) may be the major energy sources for the feline small intestine [61]. Thus, based on the dietary AA intake, cats oxidize more NEAAs than EAAs [122]. Second, there are metabolic demands for the increased use of BCAAs to synthesize aspartate, glutamate, and glutamine in extra-intestinal tissues (including skeletal muscle, white adipose tissue, and heart) in cats as in other carnivorous mammals [61]. Aspartate, glutamate, and glutamine in plasma may be the primary nutrients for ATP production in the feline liver, skeletal muscle, and kidneys, as reported for carnivorous fish [123]. Third, most NEAAs are used as glucogenic substrates in the feline liver and kidneys because the natural diet (i.e., meat) of cats provides only a small amount of glucose [124]. When cats are fed diets (e.g., some commercial foods) containing sufficient digestible carbohydrates, there is less gluconeogenesis from dietary AAs when compared with diets with insufficient digestible carbohydrates. Finally, when the content of one of 10 EAAs (Arg, Lys, His, Ile, Leu, Met, Phe, Thr, Trp, and Val; provided at 1.8 to 3.3 times NRC requirements [16]) is decreased to one-half that present in the basal diet, there is no decrease in the weight gain of young cats, but the presence of one or more NEAAs is required for their maximal growth [14].

Cats respond to nitrogen-free intake by decreasing whole-body AA oxidation, leading to reduced excretion of urinary total nitrogen, urea, and ammonia [22]. Thus, increasing the dietary content of protein from 0 to 4%, 7%, 10%, or 13% augmented the excretion of the nitrogenous metabolites in a dose-dependent manner [67]. These animals can down-regulate AA catabolism and reduce urinary nitrogen excretion when protein intake is below their requirement (20% CP). Interestingly, regarding AA metabolism, cats differ from omnivores (e.g., rats, pigs, and chickens) and herbivores (e.g., sheep) that often exhibit 3- to 4-fold greater activities of hepatic AA-catabolic enzymes in response to a high-protein diet [125]. For example, the activities of aminotransferases and urea-cycle enzymes in the liver do not differ in adult cats fed a high (70%)-CP diet and a low (17.5%)-CP diet [125]. It is likely that (1) tissues of cats express high basal levels of enzymes for AA catabolism and urea-cycle enzymes; (2) when fed a low-protein diet, cats are unable to downregulate these enzymes, but actual metabolic fluxes through the enzymes in vivo are decreased under conditions of such a nutritional state (e.g., reduced concentrations of AAs and cofactors in cells) to meet a need for conserving AAs; and (3) the activities of these enzymes are sufficient to degrade excessive AAs and remove excessive ammonia as urea in healthy cats fed high-protein diets. In support of this view, Russell et al. [126] reported that compared with adult cats fed a moderate-CP (35% of dietary ME) diet, feeding a high-CP (52% of ME) diet for 50 d increased protein oxidation by 47% but decreased fat oxidation by 37%. Likewise, increasing the dietary CP level from 9.1% to 59.6% increased protein oxidation by 507% in adult cats (Table 6). Thus, cats are metabolically more capable of adapting to high protein intake than previously realized based on enzyme activity data [125]. Cats can tolerate at least 60% dietary protein [122]. In animals (including cats), AA oxidation increases to remove excess AAs [61].

**Table 6:** Increasing dietary protein intake increases the oxidation of protein in adult cats^a^

Variable	Diets containing different levels of protein			
	Low protein	Adequate protein	Moderate protein	High protein
Body weight, kg	3.84	3.99	4.2	4.17
Dietary energy and nutrient content and food intake				
Protein content, % (as-fed basis)	9.1	17.2	32.7	59.6
Protein content, % of metabolizable energy	7.5	14.2	27.1	49.6
Fat content, % (as-fed basis)	20.9	21.0	20.9	20.9
Carbohydrate, % (as-fed basis)	65.1	57.0	41.0	13.5
Metabolizable energy, MJ/kg dry matter	20.3	20.3	20.2	20.1
Food intake, g dry matter/d	41.5	58.5	54.0	57.8
Nitrogen intake, excretion, and balance				
Nitrogen intake, g/d	0.60	1.61	2.82	5.52
Total urinary nitrogen, g/d	0.76	1.16	2.29	4.58
Urinary ammonia nitrogen, mg/d	117	152	157	198
Urinary creatinine nitrogen, mg/d	48.7	47.1	56.5	54.5
Fecal nitrogen, g/d	0.09	0.12	0.15	0.17
Nitrogen balance, g/d	−0.25	0.33	0.39	0.76
Dietary intake and the oxidation of nutrients				
Protein intake, g/d	3.8	10.0	17.6	34.5
Protein oxidation, g/d	5.7	8.6	17.1	34.6
Fat intake, g/d	8.7	12.3	11.3	12.1
Fat oxidation, g/d	12.1	10.7	13.9	9.5
Carbohydrate intake, g/d	27.1	33.3	22.2	7.8
Carbohydrate oxidation, g/d	21.6	27.1	20.3	9.2
Concentrations of amino acids in plasma				
Total amino acids, μmol/L	1827	2579	4461	5363
Leucine + Isoleucine + Valine, μmol/L	154	221	564	1286
Urinary excretion of amino acids				
EAAs (excluding taurine) and NEAAs, μmol/d	418	563	458	527
EAAs/NEAAs, mol/mol	5.4	5.7	2.6	1.7
Felinine^b^, μmol/d	388	458	1009	919

*EAAs* Nutritionally essential amino acids, *NEAAs* Nutritionally nonessential amino acids

^a^ Adapted from Green et al. [122]

^b^ Two males and two females

### Loss of muscle protein with aging

According to the current NRC [16], the requirement of non-pregnant and non-lactating adult dogs for dietary CP is similar to that of adult pigs but is only 50% of that of adult cats. Without nutritional intervention, aging dogs usually lose a substantial amount of lean body mass and gain white adipose tissue [127, 128], possibly due to reduced muscle protein synthesis, increased proteolysis, and augmented lipogenesis [61] while developing sarcopenia (defined as a loss of ≥3% lean body mass over 3 years) [128]. This can be illustrated by studies with Labrador retrievers (males plus females) that were fed a growth diet (containing 27.5% CP and 15.0 kJ ME/g diet) between 8 weeks and 3.25 years of age) and an adult diet (containing 21.2% CP and 14.8 kJ ME/g diet) between 3.25 and 14 years of age; those dogs lost 21% lean body mass between 8 and 13 years of age and had a median life span of 11.2 years [129]. Because a 15% reduction in lean body mass of animals (including dogs) impairs organic and physiological functions, and a > 30% reduction may be fatal [130], it is imperative to mitigate sarcopenia in aging dogs. To alleviate insulin resistance with aging, protein requirements should be increased by about 50% in older dogs compared with young adults [130, 131]. Protein restriction for healthy older dogs can be detrimental to their health (particularly regarding the mass and function of skeletal muscles and bones) and, therefore, should be avoided in feeding practice. To meet the dietary requirements of dogs for high-quality protein, animal-sourced foodstuffs [which provide proper ratios and adequate amounts of proteinogenic AAs as well as functional nutrients (e.g., taurine, 4-hydroxyproline, creatine, and carnosine)] can be useful ingredients for canine diets [54]. In support of this view, increasing dietary CP intake from 16% to 32% enhanced whole-body protein synthesis in both young adult (2 years of age) and aging (8 years of age) dogs [132].

Like dogs, cats lose lean body mass with age. For example, aging cats lose 34% lean body mass over an 8-year period between 7 and 15 years of age [128]. As for older dogs, older cats need adequate high-quality protein (i.e., 40% animal protein in diet; DM basis) to alleviate aging-associated reductions in the mass and function of skeletal muscles and bones [71]. Such diets should also help to improve the anti-oxidative and immune functions of senior cats. Consistent with this notion, adult cats (neutered males) needed 1.5 g protein/kg BW/d (i.e., 2.1 g/kg BW^0.75^/d) to maintain nitrogen balance but required 5.2 g protein/kg BW/d (i.e., 7.8 g/kg BW^0.75^/d) to maintain lean body mass [71]. This value is equivalent to 40% protein in the diet and greatly exceeds current NRC [16] recommendations for dietary protein requirement of adult cats. Protein-restricted diets for healthy adult cats must be avoided in feeding practice. To meet the dietary requirements of adult cats and dogs for high-quality protein, animal-sourced foodstuffs can be used to manufacture diets for these animals [54].

Dietary AA intake by cats and dogs, like other animals, depends on dietary AA content and food consumption [61]. Multiple factors should be considered in formulating diets, including endogenous AA synthesis, the digestibility and bioavailability of dietary nutrients, the presence of antinutritive factors in foodstuffs, the fermentability and quantity of dietary fiber, and interactions among food constituents [33, 61, 133]. Furthermore, requirements of cats and dogs for dietary AAs (including sulfur AAs) may critically depend on the catabolism of these nutrients by the intestinal microbiota [75, 99].

## Requirements of cats and dogs for dietary amino acids during pregnancy and lactation

Average pregnancy length in cats and dogs appears to similar (65 and 63 d, respectively), but there are differences in the patterns of both maternal and fetal weight gains between these two species [134, 135]. In dogs, maternal weight gain (almost exclusively the conceptus growth) is minimal in the first 6 weeks of gestation and increases by 25% during the last 3 weeks of gestation [134]. In cats, maternal weight gain (including both the maternal body fat gain and the conceptus growth) occurs linearly during gestation and is 43% of their pre-pregnancy BW [134]. In cats and dogs, 82% and 90% of fetal growth occurs in the last 3 weeks of gestation, respectively [134]. A deficiency of maternal dietary taurine (≤ 0.05%) causes early embryonic resorptions and fetal defects in cats and their diets must contain > 0.05% taurine for optimum pregnancy outcomes [136]. At present, little is known about the dietary requirements of pregnant dogs for taurine.

Lactation lasts approximately 7–8 weeks in cats and dogs, with peak milk production around weeks 3 to 4 [134]. There are differences in maternal weight change after parturition between cats and dogs. Specifically, after giving births, the bitch generally returns to her pre-breeding BW immediately after delivery [134]. In contrast, the queen losses BW (mainly fats) gradually to reach her pre-breeding weight at 24 d post-partum and her BW at 5 to 7 weeks post-partum is about 95% of her pre-breeding weight [137]. As in other mammals, dietary deficiencies of AAs impair milk production by lactating cats and dogs [16].

To date, dietary requirements of pregnant and lactating cats and dogs for proteinogenic AAs have not been well defined. It has been assumed that dietary CP requirements for the growth of young cats and dogs would meet their requirements for gestation and lactation [16]. However, embryos of mammals, including cats and dogs, are highly sensitive to ammonia toxicity and, therefore, maternal intakes of dietary CP and AAs should not be excessive [138, 139]. In gestating and lactating dogs, the requirement for dietary CP is 260 g/kg diet containing 4.0 kcal ME/g of the diet without dietary carbohydrate or 200 g/kg diet containing 4.0 kcal ME/g of the diet with dietary digestible carbohydrate [16]. The NRC [16] recommends that the dietary requirements of dogs or cats for CP and AAs be the same during late pregnancy and peak lactation, but it is likely that such estimates do not reflect the true requirements of the animals because of marked differences in physiological states (pregnancy versus lactation) and products (conceptus versus milk). In addition, the NRC [16] recommends substantial increases in the dietary contents of CP and most proteinogenic AAs except methionine, cysteine and tryptophan for adult dogs during late gestation and peak lactation compared with non-pregnant and non-lactating counterparts (Table 7). Disappointingly, the NRC [16] did not explain its recommended 3%, 6%, and 14% decreases, respectively, in the dietary content of cysteine, methionine and tryptophan for dogs during both late gestation and peak lactation compared with non-pregnant and non-lactating adult dogs. Such recommendations do not appear to have physiological bases and should be revised in the future.

**Table 7:** Recommended allowances of dietary AAs for post-weaning growing dogs and cats, as well as adults

Nutrient	Dogs, Amt/kg DM				Cats, Amt/kg DM				Pigs, Amt/kg DM			
	Growing^a^	Adult (NP)	LG	PL	Growing	Adult (NP)	LG	PL	6-kg BW	110-kg BW	P1G	P1LS
	NRC (2006)				NRC (2006)				NRC (2012)^e^			
ME, kcal	4000	4000	4000	4000	4000	4000	4000	4000	3778	3667	3667	3667
Crude protein, g	225	100	200	200	225	200	213	213	252	116	142	228
EAAs, g												
Arg	7.9	3.5	10.0	10.0	9.6	7.7	15	15	8.3	3.6	4.4	5.3
His	3.9	1.9	4.4	4.4	3.3	2.6	4.3	4.3	6.4	2.8	1.9	3.9
Ile	6.5	3.8	7.1	7.1	5.4	4.3	7.7	7.7	9.8	4.3	4.6	5.4
Leu	12.9	6.8	20.0	20.0	12.8	10.2	18	18	19.4	7.9	7.9	11.0
Lys	8.8	3.5	9.0	9.0	8.5	3.4	11	11	18.9	7.9	8.4	9.6
Met	3.5	3.3	3.1	3.1	4.4	1.7	5.0	5.0	5.4	2.3	2.4	2.6
Met + Cys	7.0	6.5	6.2	6.2	8.8	3.4	9.0	9.0	10.7	4.8	5.7	5.2
Phe	6.5	4.5	8.3	8.3	5.0	4.0	–	–	11.2	4.8	4.7	5.2
Phy + Tyr	13.0	7.4	12.3	12.3	19.1	15.3	19.1	19.1	17.8	7.8	8.3	10.9
Thr	8.1	4.3	10.4	10.4	6.5	5.2	8.9	8.9	11.7	5.4	6.1	6.4
Trp	2.3	1.4	1.2	1.2	1.6	1.3	1.9	1.9	3.1	1.4	1.6	1.8
Val	6.8	4.9	13.0	13.0	6.4	5.1	10	10	12.2	5.4	6.1	8.3
Taurine (Cats)	–	–	–	–	0.40	0.40	0.53	0.53	–	–		
NEAAs, g	Li and Wu^c^				Che et al. [140]^d^				Wu and Li [139]^f^			
Ala	15.2	5.63	10.6	12.1	23.3	20.1	29.1	34.9	14.4	7.99	8.91	10.5
Asn	10.6	3.95	7.06	8.46	17.1	14.8	21.5	25.8	10.5	5.75	6.46	8.53
Asp	15.2	5.63	10.6	12.1	21.0	18.3	26.5	31.8	14.7	8.17	7.88	12.1
Glu	26.6	9.88	17.6	21.2	38.4	33.3	48.3	58.0	25.6	14.1	11.5	23.4
Gln	24.0	8.92	15.9	19.1	25.5	22.1	32.0	38.4	23.8	12.6	20.7	18.3
Gly	16.9	5.82	10.4	12.5	17.3	14.9	21.6	25.9	16.2	9.07	6.20	9.58
Pro + Hyp^b^	19.0	6.27	11.2	13.4	17.8	15.4	22.3	26.8	18.6	10.2	11.5	15.8
Ser	9.30	3.46	6.18	7.42	18.1	15.6	22.6	27.1	8.74	4.87	5.81	9.24

*Amt* Amount, *BW* Body weight, *DM* Dry matter of diet, *EAAs* traditionally nutritionally essential amino acids, *Hyp* 4-hydroxyproline, *LG* Late gestation, *ME* Metabolizable energy, *NEAAs* traditionally nutritionally nonessential amino acids, *NP* Non-pregnant and non-lactating, *NRC* National Research Council, *PL* Peak lactation, *P1G* the last 24 d of pregnancy of first-parity swine, *P1LS* first-parity lactating sows

“–” data are not available

^a^ Referring to 4- to-14-week-old dogs for EAAs. The recommended allowance of dietary crude-protein for ≥14-week-old growing dogs is 175 g/kg DM

^b^ The ratio of proline to 4-hydroxyproline is 18.6:1.0, g/g

^c^ Present work. It is assumed that the recommended allowance of dietary crude-protein for adult dogs is 10%, dry matter basis

^d^ It is assumed that the recommended allowances of dietary crude-protein for growing and young adult cats are 30% and 26%, respectively, on the dry matter basis [140]. The recommended allowances of dietary EAAs for growing cats (g/kg dry matter of diet) are: Arg, 26.8; Cys, 6.3; His, 16.3; Ile, 21.0; Leu, 34.1; Lys, 36.8; Met, 12.9; Phe, 17.1; Thr, 18.9; Trp, 5.1; Tyr, 15.4; and Val, 24.3. The recommended allowances of dietary EAAs for young adult cats (g/kg dry matter of diet) are: Arg, 23.3; Cys, 6.3; His, 14.0; Ile, 18.3; Leu, 29.5; Lys, 31.9; Met, 11.3; Phe, 14.9; Thr, 16.4; Trp, 4.4; Tyr, 13.4; and Val, 21.0. The recommended allowances of dietary crude-protein for young and adult cats are 1.25 times their minimum dietary requirements for crude protein

^e^ Different breeds of swine have different body weights at young and adult ages. For the offspring of Yorkshire × Landrace sows and Duroc × Hampshire boars with a normal birth weight, 6- and 110-kg body weights correspond to approximately 3 weeks and 6 months of age. At about 18 months of age (adult), the lean-tissue weight curve of swine is flattened. The dietary requirement of adult swine for crude protein is approximately 100 g/kg dry matter

^f^ Values refer to amino acid content, rather than digestible amino acid content, in the diet

## Important roles of animal-sourced foodstuffs in providing AAs in diets for cats and dogs

### Abundant sources of both arginine and taurine in animal-sourced foodstuffs for cats and dogs

Compared with adult humans, adult dogs have a 90% greater rate of whole-body arginine catabolism and, consequently, a much higher requirement for arginine [63]. This necessitates a higher intake of good-quality protein by dogs than humans to both directly supply exogenous arginine and endogenously generate arginine from its precursor AAs (glutamine/glutamate and proline) via the intestinal-renal axis [63]. Of particular note, most dietary glutamine (~ 70%) and glutamate (~ 95%) as well as ~ 40% of dietary proline are extracted by the mammalian small intestine during their first pass into the portal vein [75] and, thus, most of these three AAs in the body are derived from endogenous syntheses. Interestingly, glutamine is the only AA in the arterial blood that is taken up by the small intestine for citrulline and arginine production; therefore, it is of nutritional and physiological importance to convert BCAAs (the source of the amino group and amide nitrogen) into glutamine in extra-hepatic tissues (primarily skeletal muscle) [61]. Because BCAAs are not formed de novo in all animals, these EAAs must be provided from high-quality and high-quantity protein. Even the same ingredient may not supply the same amount of nutrients depending on the method of food processing, and some of diet-derived small peptides can exert signaling and regulatory functions in the intestine and extraintestinal tissues [141]. Despite an endogenous synthesis of arginine, both young and adult dogs must ingest adequate arginine in diets to maintain its vital physiological functions beyond nitrogen balance [43], as noted previously. Interestingly, hydrolyzed feather is a rich source of arginine (5.83%, as-fed basis) [54]. Inclusion of hydrolyzed feather meal in dry or wet foods for dogs can meet their high requirements for arginine. In addition, even when fed a diet containing sufficient methionine and cysteine, some breeds of dogs have a limited ability to synthesize taurine due to genetic mutations as noted previously and, therefore, must be provided with adequate dietary taurine (e.g., 0.4% of dietary DM) [100].

As noted previously, cats have a very limited ability to synthesize both arginine and taurine and, therefore, must consume diets containing these two AAs to ensure normal blood flow, the proper digestion of dietary lipids and fat-soluble vitamins, and maintain health (particularly retinal, cardiac, skeletal, reproductive, and metabolic health) [14, 43]. These animals do not have preference for plant products that generally contain high amounts of carbohydrates including sweet sugars [14]. In recent years, much work has shown that animal-derived ingredients are abundant sources of both arginine and taurine for the diets of animals [54]. For example, the content of taurine in common animal-derived foods (mg/kg food, on an as-fed basis) is: blood meal, 1520; chicken by-product meal, 2096; chicken visceral digest, 1317; spray-dried peptone from enzyme-treated porcine mucosal tissues, 1638; poultry by-product meal (pet-food grade), 3884; and spray-dried poultry plasma, 2455. These foodstuffs also contain creatine that is essential for energy metabolism and anti-oxidative reactions in excitable tissues (brain and skeletal muscle) [61]. In contrast, all plant-sourced foodstuffs lack taurine and creatine [54] and, therefore, should not be fed solely to either cats or some breeds of dogs. As for dogs, hydrolyzed feather meal can be included in diets as an abundant source of both arginine and taurine for cats.

### Abundant sources of glycine, proline, 4-hydroxyproline, cysteine, and serine in animal-sourced foodstuffs for cats and dogs

Another unique feature of animal-derived products is that they contain high amounts of either collagens (e.g., meat and bone meal and poultry by-product meal) or keratins (e.g., hydrolyzed feather meal) [61]. Collagen is comprised of two-thirds of AAs as glycine, proline, and 4-hydroxyproline, whereas keratins (present in feather and hair) are also rich in these three AAs plus cysteine and serine (the immediate precursor of glycine). After feather and hair are properly hydrolyzed, their AAs are nutritionally available for both cats and dogs to use [54]. For example, the content of the most abundant AAs in chicken feather keratin (% of total AAs, mol/mol) is: glycine, 13.7; proline, 9.8; cysteine, 7.8; and serine, 14.1 [142]. Thus, hydrolyzed feather meal contains high amounts of glycine, proline, 4-hydroxyproline, cysteine, and serine (8.97%, 11.64%, 4.97%, 4.17%, and 8.92%, respectively, as-fed basis) [54]. Dietary provision of hydrolyzed feather meal can spare energy and materials that would be needed for de novo syntheses of these AAs in animals, thereby reducing energy expenditure as well as the associated production of oxidants (e.g., formaldehyde) and ammonia [61]. Of note, cysteine, glycine, and proline are crucial for the synthesis of hair proteins (e.g., cysteine-rich α-keratin and β-keratin) as are both glycine and proline for the synthesis of collagen and elastin [61]. These unique proteins maintain the normal structures and integrity of hair and connective tissue while preventing its abnormalities particularly in association with aging. Indeed, hair quality is considered by pet owners as a very important indicator of the nutritional adequacy of commercially manufactured pet foods or home-made meals [137]. Thus, hydrolyzed feather meal may be a desirable pet-food ingredient to provide nutritionally and physiologically significant AAs (including arginine, cysteine, glycine, proline, 4-hydroxyproline, and serine) [54]. This new knowledge can help to dispel the unfounded myth that poultry-sourced hydrolyzed feather meal is of little nutritive value in feeding companion animals.

### Provision of glucosamine in animal-sourced foodstuffs for cats and dogs

Glucosamine is a normal metabolite of glutamine and fructose-6-phosphate in animals [61]. Poultry meal is manufactured from raw materials containing chicken cartilage, which consists of glycosaminoglycans (including chondroitin) and proteoglycans (formed from glycosaminoglycans and protein backbones) in addition to collagens and elastins. Glycosaminoglycans are composed of N-acetylglucosamine and N-acetylgalactosamine, as well as their sulfate derivatives [61]. In the small intestine, proteoglycans are hydrolyzed to proteases (e.g., trypsin) to generate glycosaminoglycans, peptides, and AAs; the combined actions of exoglycosidase, endoglycosidase, sulfohydrolase, and hyaluronidase-like enzymes along with deacetylase release glucosamine and galactosamine from glycosaminoglycans, whereas chondroitinase and deacetylase hydrolyze chondroitin to galactosamine and glucuronic acid [61]. The aminosugars are absorbed into enterocytes and then the portal vein. Within cells, 4-epimerase converts galactosamine into glucosamine. Thus, poultry meal is a source of glucosamine for animals, including cats and dogs. Of particular note, glucosamine has anti-inflammatory and anti-oxidative effects in immunologically challenged mammalian cells by inhibiting the expression of inducible NO synthase and excessive NO production [143]. This may explain why glucosamine plus chondroitin has been used to effectively treat dogs (particularly elderly dogs and working dogs) [144] and cats [145] with joint pain or osteoarthritis. Research is warranted to define the efficacy of dietary supplementation with poultry meal in improving the health of cat and dog joints.

### Improvement of immune responses in cats and dogs

AAs are essential for immune responses in all animals (including cats and dogs) through a plethora of mechanisms, such as the syntheses of proteins (including antibodies and cytokines) and glutathione (a potent anti-oxidative tripeptide consisting of glycine, cysteine, and glutamate), as well as the killing of pathogens via production of NO from arginine and of chlorotaurine and bromotaurine from taurine [61]. Supplementing arginine to a low-protein (23% CP) [146] or high-protein (60% CP) [147] diet has beneficial immunomodulating effects in cats. In addition, intravenous administration of alanyl-glutamine to dogs undergoing a treatment with methylprednisolone sodium succinate enhanced phagocytic capacity and respiratory burst activity of leukocytes [148]. Currently, there is a global pandemic of COVID-19 caused by the severe acute respiratory syndrome coronavirus 2 that can also infect dogs [149] and cats [150]. Adequate AA nutrition [e.g., sufficient provision of AAs (such as lysine, cysteine, methionine, tryptophan, glycine, and proline) that are abundant in animal proteins but are relatively low in plant proteins] may be crucial for improving both innate and acquired immune systems to mitigate risk for infection in the animals. In addition, some animal-sourced foods, such as spray-dried animal plasma [151] and spray-dried egg products [152] contain a large amount of immunoglobulins and directly contribute to neutralizing the pathogens that invade the body. Furthermore, spray-dried animal plasma provides other functional molecules, such as albumin, essential fatty acids, B-complex vitamins, and minerals such as calcium, phosphorus, sodium, chloride, potassium, magnesium, iron, zinc, copper, manganese, and selenium [153, 154]. This animal-sourced foodstuff is also a useful binder in canned pet food products due to its high content of globulins and fibrinogen as well as its desired physicochemical properties [155]. Finally, animal plasma products are highly palatable to both cats and dogs [156]. Thus, animal-derived ingredients used in dry or wet pet foods can help to improve the immune responses and health of all companion animals by providing not only AAs but also other essential nutrients (e.g., macro- and micro-minerals).

## Conclusions and perspectives

Both cats and dogs are carnivores from the taxonomical order Carnivora. During evolution, domestic dogs have adapted to omnivorous diets that contain both taurine-rich meat and starch-rich plant ingredients, while domestic cats remain obligate carnivores. Thus, dogs differ from cats in many aspects of AA nutrition and metabolism, and dogs can thrive on taurine-free vegetarian diets supplemented with non-taurine nutrients that are inadequately synthesized de novo or absent from plants. Much evidence shows that there are marked differences in both qualitative (i.e., presence or absence) and quantitative (i.e., amounts) requirements for protein and certain AAs (arginine, taurine, methionine, and cysteine, as well as NEAAs) between cats and dogs. In comparison with swine [53, 139], recommended minimum requirements and allowances of dietary EAAs for growing and adult cats and dogs are summarized in Table 7. We suggest that companion animals have dietary requirements for NEAAs as do other mammals due to insufficient synthesis de novo. Cats have greater endogenous nitrogen losses, as well as higher requirements for dietary protein (including arginine) and taurine than do dogs and, therefore, should not be fed dog foods. Because the composition of the milk of both cats and dogs differ from that of farm mammals, young pets should not be fed replacer diets formulated based on goat or cow milk. As companion animals lose tremendous amounts of lean body mass with aging, their diets should contain adequate levels of high-quality protein, which may be much greater than the current AAFCO [157] and NRC [16] recommendations to support muscle protein synthesis and mitigate muscle loss.

Effects of an excessive intake of a single AA in cats [140] and dogs [108] may be different, depending on dietary intakes of other AAs. We are not aware that comparisons of the metabolism or dietary requirements of any nutrients between modern breeds of cats and dogs were made in the same experiment. Nonetheless, the fundamental knowledge of nutrient metabolism in cats and dogs is essential for guiding their feeding, as well as food manufacturing. In practice, animal-sourced foodstuffs provide proper ratios and amounts of all proteinogenic AAs as well as large amounts of functional nutrients (e.g., taurine, 4-hydroxyproline, creatine, and carnosine), lipids, and minerals [61]. In addition, feather meal can be used as an ingredient or supplement in dry or wet foods for cats and dogs to meet their high requirements for both arginine and taurine [54]. Animal-derived ingredients alone or in combination are abundant sources of both proteinogenic AAs and taurine for adequate nutrition and metabolism in cats and dogs to optimize their growth, development, health, and well-being.

## Data Availability

This manuscript reviews published work and does not contain new experimental data.

## Abbreviations

AA: Amino acid
AAFCO: Association of American Feed Control Officials
BCAA: Branched-chain amino acid
BW: Body weight
CP: Crude protein
DM: Dry matter
EAA: Nutritionally essential amino acid
ME: Metabolizable energy
NEAA: Nutritionally nonessential amino acid
NO: Nitric oxide
NRC: National Research Council
P5C: Pyrroline-5-carboxylate
